# Potential drug-drug interactions in hospitalized patients with acute coronary syndrome at a tertiary care hospital in upper Egypt

**DOI:** 10.3389/fphar.2026.1761758

**Published:** 2026-04-14

**Authors:** Ayad A. Ghabesha, Sahar B. Hassan, Hosam Hasan-Ali, Mohamed M. M. Abdel-Latif

**Affiliations:** 1 Ministry of Health and Population, Aden, Yemen; 2 Department of Clinical Pharmacy, Faculty of Pharmacy, Assiut University, Assiut, Egypt; 3 Department of Cardiovascular Medicine, Faculty of Medicine, Assiut University, Assiut, Egypt

**Keywords:** acute coronary syndrome, drug therapy, drug-drug interactions, drug-related problem, Egyptian

## Abstract

**Background:**

Potential drug-drug Interactions (pDDIs) are a serious concern in cardiovascular patients and are responsible for most adverse drug reactions and drug-related hospital admissions. Clinically significant pDDIs may injure patients, create adverse outcomes, and impact healthcare costs. Therefore, pDDIs control is critical for the improvement of prescription drug safety.

**Aim:**

The present study aimed to assess potential pDDIs among cardiovascular patients at a Tertiary Care Hospital in Upper Egypt.

**Methods:**

A prospective observational study was performed on acute coronary syndrome (ACS) patients attending Assiut University Heart Hospital. The Lexi-Interact online was used to assess pDDIs. Multiple logistic regression was used to identify the factors associated with pDDIs.

**Results:**

A total of 371 patients attending Assiut University Heart Hospital were recruited for the study. A total of 2,504 pDDIs were detected among the patients. The mean number of drugs prescribed per patient was 7.78 ± 1.73. Class C, D, and X DDIs were found in 74.76%, 10.02%, and 0.04%, respectively. Aspirin and ticagrelor were the most prevalent pDDIs under category D (232, 92.4%). A combination of ramipril and aspirin was the most frequently encountered interacting pair in the class C (281, 17.2%). STEMI patients, length of hospital stay, and a higher number of prescribed drugs were significantly associated with the occurrence of pDDIs, according to multiple logistic regression.

**Conclusion:**

Patients with ACS were frequently exposed to pDDIs with the highest drug-related problems, treatment failure, and negative consequences. Therefore, clinical pharmacists ought to be more involved in managing complex drug regimens and improving therapeutic outcomes.

## Introduction

Acute coronary syndrome (ACS) is characterized by atherosclerotic plaque breakdown and coronary arterial blockage. ACS includes a ST-segment elevation myocardial infarction (STEMI), unstable angina (UA), and NSTEMI (Kumar and Cannon, 2009; Boden et al., 2012; Pejcic et al., 2023). Modern care of ACS depends on guideline-directed medical therapy that uses more than one type of medication.

The European Society of Cardiology and the American Heart Association (ESC/AHA) now recommend starting dual antiplatelet therapy (aspirin plus a P2Y12 inhibitor) as soon as possible, as well as anticoagulation when necessary, high-intensity statins, beta-blockers, and renin–angiotensin–aldosterone system inhibitors, such as angiotensin-converting enzyme inhibitors (ACEIs) or angiotensin receptor blockers (ARBs) (Timmis et al., 2023; Byrne et al., 2024; Rao et al., 2025). This evidence-based polypharmacy greatly increases survival and lowers the number of ischemic events that happen again, but it also raises the risk of drug-drug interactions (Timmis et al., 2023).

Patients with ACS who are in the hospital are especially at risk for the potential drug-drug interactions (pDDIs) because their antithrombotic regimens are complicated, they often take cardiovascular drugs with restricted therapeutic indices, and they have several other health problems. Even while pDDIs are important in clinical practice, there is not a lot of complete data on how often they occur and how they affect ACS patients at Assiut University Heart Hospital.

Identifying and characterizing pDDIs in this high-risk population may assist physicians in forecasting adverse medication events, enhancing prescribing practices, and reducing preventable hospital readmissions. The present investigation aimed to assess the prevalence and patterns of pDDIs among hospitalized patients with ACS and to identify the most common pDDIs within this population.

## Patients and methods

### Study design and setting

A prospective observational study was performed on patients with ACS. Patients were admitted to Assiut University Heart Hospital in Assiut, Egypt, from 1 April 2022, to 31 March 2023.

### Ethical approval

The research ethics committee of the Faculty of Medicine at Assiut University authorized the investigation. The research design received approval from the Institutional Review Board of the Faculty of Medicine, Assiut University (IRB number 17200697). The study was conducted in accordance with the principles of the Helsinki Declaration. The Clinicaltrial.gov registration ID was: NCT07008794. All participants provided written informed consent before the commencement of the study, and patient confidentiality was rigorously upheld throughout the research.

### Sample size

G-Power estimated a sample size of 168, with 84 cases per category. The following factors were used to calculate the sample size: The difference in the decrease in LDL-C concentration between rosuvastatin and atorvastatin resulted in a moderate effect size of 0.5 (Javed et al., 2020). The test’s power was 80%, the allocation ratio was N2/N1 = 1, and the confidence level was 95%. The final sample size was set at 100 patients per group, with an additional 15% to account for potential dropouts. The study included 371 patients to minimize lost to follow-up.

### Inclusion and exclusion criteria

Adult patients (≥18 years) with a confirmed diagnosis of ACS, encompassing STEMI, NSTEMI, or UA, who were admitted as inpatients to Assiut University Heart Hospital, Assiut, Egypt, from April 2022 to March 2023, were eligible for inclusion. Patients referred for evaluation or exclusively handled in outpatient clinics were excluded. The research was executed in a single center.

### Data collection

The demographic and clinical information were collected from patients’ medical records. It includes their age, sex, comorbidities, diagnosis, hospital stay, and a full list of all the drugs they used. Patients were observed from admission to the hospital until discharge to ensure a comprehensive record of all prescribed drugs. The World Health Organization Collaborating Centre’s Anatomic Therapeutic-Chemical (ATC) Drug Classification (20th Ed., 2017) was employed to classify drugs.

### Identification and classification of potential drug-drug interactions

Using the Lexi-Interact online (Lexi-Comp Inc., Hudson, United States), the drug regimens for pDDIs were evaluated. Easy access to Lexi-Interact is acknowledged as a benefit of computerized software. Lexi-Interact concisely furnishes data regarding the severity, reliability, and risk of pDDIs. It also provides detailed recommendations for preventing and managing pDDIs (module, 2024). The Lexicomp program was used due to the unavailability of certain drugs, including trimetazidine, gliclazide, and nicorandil, in alternative drug interaction databases.

The principal investigator used Lexi-Interact to carefully review all medical profiles for pDDIs. This was done using a systematic assessment procedure to ensure the classification was consistent. Even though there was not an independent duplicate review, the systematic approach was intended to ensure the methods remained precise and reliable.

### Categorization of pDDIs

This database categorizes pDDIs into five risk ratings based on their clinical significance: A, B, C, D, and X. C, D, and X were identified as potential clinically pertinent DDIs in most studies. Reliability is classified as excellent, good, fair, and poor based on the integrity of the evidence (Table 1) (module. 2024).

**TABLE 1:** Definitions of risk, reliability, and severity ratings for DDIs by Lexi-Interact software (module. 2024).

Classification	Definition
Risk rating	The level of urgency and actions needed to respond to DDIs
A	No known interaction
B	No action needed
C	Monitor therapy
D	Consider therapy modification
X	Avoid combination
Reliability rating	The quantity and nature of evidence
Excellent	Multiple clinical trials or a single clinical trial plus more than two case reports
Good	Single randomized clinical trial plus fewer than two case reports
Fair	More than two case reports or fewer than two case reports, plus other supporting data or a theoretical interaction based on known pharmacology
Poor	Theoretically, the interaction may occur, but reports are very limited, such as a few case reports
Severity rating	Qualify the reported or possible magnitude of DDI outcomes
Major	The effects of DDIs might be life-threatening or cause permanent damage
Moderate	Patients with DDIs may require additional care
Minor	The effects of DDIs may be tolerable and need no medical interventions

DDIs, drug-drug interactions.

The severity indicators comprise three categories: major, moderate, and minor. Table 1 provides the definitions of risk rating, reliability rating, and severity rating from the Lexi-Interact database (Moradi et al., 2020). Reliability and severity ratings were sought for categories C, D, and X to conduct this investigation. The Lexi-Interact monograph was subsequently addressed with clinical implications and management techniques. pDDIs were detected and classified based on the profile of drugs that were prescribed (Table 1) (module. 2024).

The following categories were used to categorize pDDIs according to their severity: (1) major (severe), the effects are potentially life-threatening or capable of causing permanent damage; (2) moderate, the effects may cause deterioration in patients’ clinical status and additional treatment or extension of hospital stay; (3) minor (mild), the effects are typically mild; and (4) contraindication, the consequences may be bothersome or unnoticeable but should not significantly affect the final therapeutic outcome (Table 1) (module. 2024).

The mechanism used to categorize pDDIs is as follows: (a) pharmacokinetic, (b) pharmacodynamic, and (c) unknown classifications. Further classification of pharmacokinetic pDDIs was done by determining if they were an increase or a decrease in the following areas: (a) absorption, (b) distribution, (c) metabolism, and (d) excretion. Further classification of pharmacodynamic pDDIs included the following categories: (a) synergistic or (b)antagonistic, and (c) additive (Palleria et al., 2013).

### Statistical analysis

Data analysis was performed utilizing version 26 of IBM SPSS Statistics (IBM Corp., Armonk, New York, USA). Categorical data were presented as percentages and frequencies, and the Chi-square test or Fisher’s exact test was used. For data that followed a normal distribution, continuous variables were shown as the mean plus or minus the standard deviation (SD). If the data did not fit a normal distribution, the median and the range were used to show them. The Mann–Whitney U test for nonparametric variables and the independent-samples Student’s t-test for parametric variables to compare groups. Multiple logistic regression was used to identify the factors associated with pDDIs. A P-value of less than 0.05 waconsidered s statistically significant.

## Results

### Patient characteristics

A total of 371 patients’ case prescriptions were studied. Table 2 shows the demographic data of the studied patients. The mean age was 58.7 ± ± 10.94 years (range 24.0–89.0). The study included 287 males (77.4%). The mean age in the study was 58.71 ± 10.94 years. Our findings showed that 327 patients (88.1%) had STEMI, 33 (8.9%) had NSTEMI, and 11 (3.0%) had UA among the ACS patients.

**TABLE 2:** The study patients’ characteristics (n = 371).

Patients characteristics	n = 371	%
Age: (years)		
Mean ± SD	58.71 ± 10.94	
Range	24.0–89.0	
Sex		
Male	287	77.4%
Female	84	22.6%
Diagnosis		
STEMI	327	88.1%
NSTEMI	33	8.9%
UA	11	3.0%
Presence of chronic diseases		
No	86	23.2
One	127	34.2
Two	102	27.5
Three or more	56	15.1
No. of chronic diseases (median and range)	1 (1–5)	
Comorbidities		
Hypertension	179	48.2%
Dyslipidemia	141	38.0%
Diabetes mellitus	132	35.6%
Previous IHD	35	9.4%
Chronic kidney diseases	5	1.3%
Length of hospital stay		
2 days	149	40.2%
3 days	138	37.2%
>3 days	84	22.6%
Total no. of medications for all patients	2,888	
No. of drugs prescribed per patient	7.78 ± 1.73 (3–15)	
Number of drugs administered at the hospital		
<7	87	23.5%
7	105	28.3%
>7	179	48.2%

Data are expressed as mean ± standard deviation or number (percent).

Abbreviations: CKD, chronic renal diseases; IHD, ischemic heart diseases; NSTEMI, non-ST, segment elevation myocardial infarction; STEMI, ST-segment elevation myocardial infarction; UA, unstable angina.

Many patients had comorbidities: 179 patients had hypertension (48.2%), followed by dyslipidemia (38.0%), type 2 DM (35.6%), and previous ischemic heart diseases (IHD) (9.4%). The median number of comorbidities was 1 (range 1–5). The median hospital stay was 3 days (range, 2.0–15.0 days). Table 2 explains the usual characteristics of the 371 patients. Two thousand eight hundred eighty-eight medications were prescribed during hospitalization, averaging 7.78 ± 1.73.

### Prevalence and characteristics of potential drug-drug interactions

There were 2,504 pDDIs, which were detected among 371 patients. One hundred and seventy-seven patients had fewer than seven pDDIs (47.7%), while one hundred and twelve individuals had seven pDDIs (30.2%). On the other hand, 82 patients were found to have seven or more pDDIs. The pDDIs had a median number of 6.0, with a range of 1.0–24.0 (Table 3).

**TABLE 3:** Potential drug-drug interactions characteristics in the studied patients (n = 371).

Characteristics of pDDIs	Total no. of pDDIs n = 2,504	%
Mean and median (Range)of pDDI per patient	7 ± 3.8 6 (1–24.0)	
Number of PDDI in 371 patients with PDDIs		
<7	177	47.7
7	112	30.2
>7	82	22.1
Risk rating		
B	380	15.18
C	1872	74.76
D	251	10.02
X	1	0.04
Reliability rating		
Good	774	30.91
Fair	1716	68.53
Poor	14	0.56
Severity rating		
Major	495	19.77
Moderate	1,620	64.70
Minor	389	15.54
Mechanism		
Pharmacodynamic	1732	69.17
Pharmacokinetic	492	19.65
Unknown	280	11.18
Pharmacodynamic subtypes	n = 1732	​
Addition	1,264	72.98
Antagonist	380	21.94
Synergism	88	5.08
Pharmacokinetic subtypes	n = 492	​
Absorption	6	1.22
Distribution	175	35.57
Metabolism	303	61.59
Excretion	8	1.63

pDDIs, potential drug -drug interactions.

### Classification of drug-drug interactions based on risk rating

A total of 2,504 drug pairs were examined, and most of them were identified as belonging to class C (n = 1872, 74.76%). Class B (n = 380, 15.18%), class D (n = 251, 10.02%), and class X (n = 1, 0.04%) emerged as the following most common classifications (Table 3).

### Classification of drug-drug interactions based on reliability rating

Approximately 69% of pDDIs were classified as fair-type (n = 1716, 68.53%), followed by good-type (n = 774, 30.91%) and poor-type (n = 14, 0.56%) according to the reliability rating (Table 3).

### Classification of drug-drug interactions based on severity

It was primarily determined that the severity of pDDIs was classified as minor (389 pDDIs, 15.54%), moderate (1,620 pDDIs, 64.70%), and major (495 pDDIs, 19.77%) (Table 3).

### Classification of drug-drug interactions based on mechanism

Our research observed 492 pharmacokinetic interactions, accounting for 19.65% of the total. Of these, the most significant cause (n = 175, 35.57%) was distribution, while 303 pDDIs (61.59% of the total) were attributed to metabolism, 8 pDDIs (1.63%) were attributive to excretion, and 6 pDDIs (1.22%) were absorption. A total of 1,732 pDDIs were discovered to have pharmacodynamic interactions, of which 1,264 (72.98%) were additive, 380 (21.94%) were antagonistic, and 88 (5.08%) were synergistic. 280 (11.18%) was an unknown mechanism (Table 3).

### Most commonly detected drug pairs based on risk rating

Aspirin with ticagrelor was the most common in class D (n = 232, 92.4%), followed by dapagliflozin with Insulin (n = 12, 4.8%), dapagliflozin with gliclazide (n = 4, 1.6%), dapagliflozin with glibenclamide/metformin (n = 2, 0.8%), and dapagliflozin with vildagliptin/metformin (n = 1, 0.4%). Within group C, concurrent usage of ramipril with acetylsalicylic acid was common (n = 281, 15.0%), followed by drugs that increase bleeding risk (Acetylsalicylic acid with clopidogrel (n = 125, 7.6%), and rosuvastatin with ticagrelor (n = 114, 6.1%). There was only one X DDI between moxifloxacin and amiodarone (Table 4).

**TABLE 4:** Most frequently occurring pDDI classified according to risk rating (n = 2,504).

Drug pairs	No.	%	Severity	Documentation	Risk rating	Mechanism	Potential sequence	Management strategies
Class X	**n = 1**		​	​	​	​	​	​
Amiodarone Moxifloxacin	1	100	Major	Fair	X	PD	Prolonging the QT	Avoid combination
Class D	**n = 251**		​	​	​	​	​	​
Aspirin Ticagrelor	232	92.4	Major	Fair	D	PD	Bleeding	Monitor signs of bleeding
Dapagliflozin insulin	12	4.8	Moderate	Fair	D	PD	Hypoglycemia	Consider a decrease in insulin dose and monitoring blood glucose
Dapagliflozin gliclazide	4	1.6	Moderate	Fair	D	PD	Hypoglycemia	Decrease in sulfonylurea dose when initiating therapy
Dapagliflozin glibenclomide metformin	2	0.8	Moderate	Fair	D	PD	Hypoglycemia	Consider a decrease in sulfonylurea dose and monitoring blood glucose
Insulin vildagliptin metformin	1	0.4	Moderate	Fair	D	PD	Hypoglycemia	Consider a decrease in insulin dose and monitoring blood glucose
Class C	**n= 1,872**		​	​	​	​	​	​
Aspirin ramipril	281	15.0	Moderate	Fair	C	PD	Nephrotoxic and**↓**Hypotensive effect	Monitoring creatine and BP
Aspirin clopidogrel	125	6.7	Moderate	Fair	C	PD	Bleeding	Monitor signs of bleeding
Rosuvastatin ticagrelor	114	6.1	Moderate	Good	C	PK	Myopathy	Monitor CK and LFT
Aspirin enoxaparin	111	5.9	Major	Good	C	PD	Bleeding	Monitor signs of bleeding
Atorvastatin ticagrelor	109	5.8	Moderate	Good	C	PK	Myopathy	Monitor CK and LFT
Clopidogrel pantoprazole	106	5.7	Major	Fair	C	PK	Decrease antiplatelet effect and therapeutic	Monitoring INR
Clopidogrel enoxaparin	98	5.2	Moderate	Fair	C	PD	Bleeding	Monitor signs of bleeding
Enoxaparin ramipril	69	3.7	Moderate	Fair	C	PD	Hyperkalemia	Monitor serum K
Aspirin dapagliflozin	69	3.7	Moderate	Fair	C	PD	Hypoglycemia if 3 g of aspirin per day	Monitoring blood glucose
Aspirin tirofiban	62	3.3	Moderate	Fair	C	PD	Bleeding	Monitor signs of bleeding
Clopidogrel rosuvastatin	61	3.3	Moderate	Good	C	PK	Myopathy	Monitor CK and LFT
Ticagrelor tirofiban	53	2.8	Moderate	Fair	C	PD	Bleeding	Monitor signs of bleeding
Aspirin Insulin	50	2.7	Moderate	Fair	C	PD	Hypoglycemia if 3 g of aspirin/day	Monitoring blood glucose
Bisoprolol dapagliflozin	41	2.2	Moderate	Fair	C	PD	Mask hypoglycemia	Monitoring blood glucose
Nicorandil ramipril	30	1.6	Moderate	Fair	C	PD	Hyperkalemia hypotension	Monitor serum K and BP

Abbreviations: AD, addition; Ant, antagonist; BP, blood pressure; CK, creatinine kinase; GIT, gastrointestinal; INR, international normalized ratio; K, potassium; LFT, liver function test; PD, pharmacodynamic; PK, pharmacokinetic; PPI, proton pump inhibitor; Sy, synergistic. The bold numbers indicate the total number in each class.

### Drug types according to ATC classification and their prescription frequency

**FIGURE 1:**
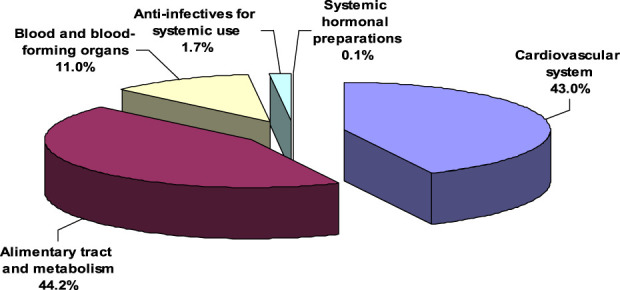
Drug types according to ATC Classification and their prescription frequency.

The frequency was calculated by multiplying each drug in the pairs by the number of frequencies. In this study, the total incidence of pDDIs was 5,138, out of 43.03% (n = 2,211) observed in the cardiovascular system, 44.2% (n = 2,271) in the alimentary tract and metabolism,10.98% (n = 10,69) in the organs responsible for blood and blood-forming organs, 1.67% anti-infectives for systemic use (n = 68), and 0.12% systemic hormonal preparations (n = 6) (Table 5; Figure 1).

**TABLE 5:** Drug types according to ATC Classification and their prescription frequency.

Treatment	n = 5,138	(%)
Alimentary tract and metabolism (A)	2,271	44.2
Dipeptidyl peptidase 4 (DPP-4) inhibitor	2059	90.7
Biguanide	89	3.9
Insulin	42	1.8
Proton pump inhibitors	32	1.4
SGLT2	26	1.1
Sulfonylurea	23	1.0
Cardiovascular system (C)	2,211	43.03
Loop diuretics	847	38.3
ARBs	644	29.1
ACEIs	303	13.7
Aldosterone receptor antagonist	166	7.5
Beta-blockers	52	2.4
Other cardiac preparation	47	2.1
Statin	37	1.7
Nitrate	34	1.5
Thiazide	25	1.1
Inotropic	24	1.1
Calcium channel blockers	19	0.9
Anti-arrhythmia	13	0.6
Blood and blood-forming organs (B)	564	10.98
Antiplatelets	493	97.8
Anti-coagulants	47	9.3
Iron	19	3.8
Minerals	5	1.0
Anti-infectives for systemic use (J)	86	1.67
Inhaled anti-infective	47	54.7
Quinolone antibacterial	19	22.1
Triazole derivatives	19	22.1
Cephalosporins	1	1.2
Systemic hormonal preparations (H)	6	0.12
Corticosteroids for systemic use	6	100

Abbreviations: ACEIs; angiotensin-converting enzyme inhibitors, ARBs; angiotensin receptors blockers, SGLTI; sodium glucose transporter inhibitors.

### Risk rating of PDDIs in relation to various risk factors

Table 6 shows the risk ratings of PDDIs across various risk factors. Although there was no significant sex variance, patients in the D and X risk groups were significantly younger than those in the B and C categories (P < 0.001). In the B and C group, diabetes and hypertension were far more common than in the D and X group, which had dyslipidemia. The B and C group had a considerably higher history of previous IHD (P < 0.001). According to clinical presentation, the D and X group had a higher prevalence of STEMI, while the B and C group had a higher prevalence of NSTEMI (P < 0.001). Despite no difference observed in EF regarding the risk rating, KILLIP classifications varied statistically. Furthermore, the B and C group received significantly more prescribed medicines length of the hospital stay (P < 0.001).

**TABLE 6:** Risk rating of PDDIs in relation to various risk factors.

Variables	Risk rating				P-value
	D and X (n = 608)		B and C (n = 1908)		
	No.	%	No.	%	
Age (years)					
Mean ± SD	57.46 ± 10.59		59.84 ± 10.82		0.000^b^
Sex					
Male	478	78.6%	1,427	74.8%	0.055^a^
Female	130	21.4%	481	25.2%	​
BMI					
Mean ± SD	25.95 ± 3.34		26.34 ± 3.69		0.022^b^
Hypertension	284	46.7%	996	52.2%	0.018^a^
Diabetic	230	37.8%	873	45.8%	0.001^a^
Dyslipidemia	257	42.3%	715	37.5%	0.034^a^
CKD	12	2.0%	49	2.6%	0.407^a^
Previous IHD	36	5.9%	215	11.3%	0.000^a^
No. of comorbidities					
Median (range)	1.0 (0.0–4.0)		1.0 (0.0–4.0)		0.001^c^
Diagnosis					
STEMI	582	95.7%	1,547	81.1%	0.000^a^
NSTEMI/UA	26	4.3%	361	18.9%	​
EF					
Mean ± SD	45.99 ± 7.77		45.59 ± 10.09		0.382^b^
KILLIP					
Median (range)	1.0 (1.0–4.0)		1.0 (1.0–4.0)		0.000^c^
Hospital stays (days)					
Median (range)	3.0 (2.0–12.0)		3.0 (2.0–15.0)		0.000^c^
No. of drugs					
Mean ± SD	7.58 ± 1.68		8.83 ± 1.92		0.000^b^

The number is not cumulative because the patient can have more than one risk factor and pDDIs.

^a^ Generated using Chi-square test or Fisher’s Exact test.

^b^ Generated using an independent Samples t-test.

^c^ Generated using Mann–Whitney U test.

Abbreviations: BMI; body mass index, CKD; chronic renal disease, EF; ejection fraction, IHD; ischemic heart disease, NSTEMI; non-ST-segment elevation myocardial infarction, STEMI; ST-segment elevation myocardial infarction, UA; unstable angina.

### Multivariate analysis of factors correlated with pDDIs in ACS patients

In Table 7, the Multiple logistic regression was used to identify the factors associated with pDDIs. The results showed that patients diagnosed with STEMI were significantly more likely to be classified in the D and X high-risk group (OR = 3.734; 95% CI: 2.437–5.721; P < 0.001). On the other hand, a longer hospital stay (OR = 0.855; 95% CI: 0.793–0.923; P < 0.001) and a higher number of prescription drugs (OR = 0.697; 95% CI: 0.649–0.749; P < 0.001) were also linked to a lower chance of being classified as D and X. Other variables, including age, BMI, hypertension, diabetes, dyslipidemia, a previous of IHD, and Killip class, did not directly influence the incidence of pDDIs.

**TABLE 7:** Multiple logistic regression analysis for risk factors of severity.

Variables	P-value	OR	95% C.I.	
			Lower	Upper
Age (years)	0.083	0.992	0.982	1.001
BMI	0.396	0.988	0.960	1.016
Hypertension	0.250	0.887	0.724	1.088
Diabetic	0.427	1.093	0.878	1.360
Dyslipidemia	0.140	1.162	0.952	1.420
Previous IHD	0.192	0.768	0.517	1.142
STEMI	0.000*	**3.734**	2.437	5.721
KILLIP	0.329	1.105	0.904	1.352
Hospital stay (Days)	0.000*	**0.855**	0.793	0.923
No. of drugs	0.000*	**0.697**	0.649	0.749

Abbreviations: BMI; body mass index; IHD, ischemic heart disease; STEMI, ST-segment elevation myocardial infarction. Bold numbers indicate the significant.

## Discussion

DDIs are a significant public health concern that often makes the clinical management of patients more difficult, particularly in the case of multi-morbidity patients treated for ACS. This study assessed the frequency of pDDIs and the variables that caused increased interactions among ACS cases hospitalized at Assiut University Heart Hospital in Assiut, Egypt.

The frequency of pDDIs was 100% among ACS case studies. Akbar et al. (2021) reported a 100% frequency of pDDIs (Akbar et al., 2021). Raslan et al. (2018) reported a pDDI frequency of 96.8%. (Raslan et al., 2018). Moreover, a close frequency of 91.6% (Murtaza et al., 2016). In contrast, several studies showed a higher frequency of pDDIs in India (30.67%) (Patel et al., 2011), Ethiopia (74.4%) (Diksis et al., 2019), Nepal (62.5%) (Sharma et al., 2014), Serbia (83.1%) (Kovačević et al., 2017), and Iran (43.4%) (Namazi and Moosavi, 2012).

This result may be explained by the fact that around half of the patients had multiple comorbidities, as demonstrated in Table [2], which illustrates a considerable increase in DDIs with increasing comorbidities, particularly ACS. Other research has reached the same conclusion, which is that individuals who suffer from cardiovascular illness are at an increased risk for DDIs (Kovačević et al., 2017; Nusair et al., 2020). In addition, the presence of polypharmacy medication in hospitalized patients is an essential aspect that helps to explain the high rate of pDDIs that was documented in this study. Additional factors contributing to a high rate of pDDIs include the time spent in the hospital (Moura et al., 2009; Diksis et al., 2019).

In this study, 2,504 pDDIs were detected from 371 prescriptions, with an average of 7 ± 3.8. Approximately 52.3% of patients experienced at least seven pDDIs. An investigation of a similar study detected that 78.2% of the cases contained at least one pDDI (Ahmad et al., 2015). Differences in the circumstances of the patients who were included in the research and the amount of treatment they received, as well as differences in the methodologies that were utilized, notably the software that was used to identify these DDIs, may be the cause of the heterogeneity that was shown in the findings of the various investigations (de Oliveira et al., 2021).

Interactions of moderate severity (n = 1,620, 64.70%) were the most frequently identified severity rating pDDIs. Comparable findings have noted moderate severity in previous investigations performed in Egypt and Pakistan (72.5% and 74.1%) (Raslan et al., 2018; Akbar et al., 2021). Similarly, in studies performed elsewhere, moderate severity was the most observed pDDIs (Patel et al., 2011; Sharma et al., 2014; Murtaza et al., 2016; Kovačević et al., 2017; Khan et al., 2019; Akbar et al., 2021; Wang et al., 2022).

In this study, many pDDIs experienced class C (n = 1872, 74.76%), followed by class B (n = 380, 15.18%), class D (n = 251, 10.02%), and class X (n = 1, 0.04%). Abdelkawy et al. (2023) found that the majority of pDDIs were categorized as C, followed by D, and then X, each representing 48.2%, 28.3%, and 23.4% of all interactions, respectively (Abdelkawy et al., 2023). Akbar et al. (2021) found that class C pDDIs were most common (n = 1971, 70.72%), followed by class D (n = 582, 20.88%), class B (n = 204, 7.32%), and class X (n = 30, 1.08%) (Akbar et al., 2021).

Moreover, Kovačević et al. (2017) observed that type C was the most common pDDI (2,960), followed by D (n = 137) and X (n = 17) (Kovačević et al., 2017).

The fair reliability rating was the most common reliability rating of pDDIs in this study (68.53%), followed by good (30.91%) and poor (0.56%). A study’s findings revealed that a fair level was observed in 45.5% of all noted pDDIs, whereas levels of good and excellent documentation were observed in 35.8% and 18.7% of all pDDIs, respectively (Kalash et al., 2023). Additionally, Akbar et al. (2021) found that fair (77.61%), followed by good (17.87%) and excellent (3.77%), were the most common reliability ratings (Akbar et al., 2021). Moreover, Ismail et al. (2012) stated that, among 1,120 pDDIs, the scientific evidence was fair (507, 45.3%) or good (470, 42.0%) (Ismail et al., 2012). In contrast, Kovačević et al. (2017) reported good results in 57.8% of pDDIs, followed by fair in 34.3%, excellent in 5.7%, and poor in 2.3% (Kovačević et al., 2017).

In this study, a pharmacodynamic interaction was noted in 1,732 (69.17%) cases, whereas a pharmacokinetic interaction was observed in 492 (19.65%) and 280 (11.18%) cases; the putative mechanism was unknown in these latter cases. Comparable results were found in a study. Egger et al. (2003) found that pharmacodynamics was the most prominent factor in 296 cases (73.6%), followed by pharmacokinetics in 94 cases (23.6%), and that the putative mechanism was unknown in 12 cases (4.0%) (Egger et al., 2003).

The pharmacokinetic component accounted for 702 (34.1%) of the total, whereas the pharmacodynamic component accounted for 1,150 (54.9%), according to research by Kalash et al. (2023). Furthermore, researchers found that pharmacokinetic (69.3%) and unknown (1.1%) were the most common types of studies (Faisal et al., 2018).

The majority of DDIs were found to be pharmacodynamic (73%), with a small percentage being pharmacokinetic (24%), and the remaining 3% being unknown (Barot et al., 2015). Moreover, it has been reported that pharmacodynamic interactions were the dominant mechanism (85.24%) (Permatasari et al., 2024). Contrary to our findings (Alkhawlani et al., 2024), pharmacokinetics accounted for 52.2% of pDDIs, followed by pharmacodynamics at 47.2% and an unknown mechanism at 10.8% (Alkhawlani et al., 2024).

In this study, out of the 1732 pharmacodynamic pDDIs (69.17%) analyzed, 88 (5.08%) were found to have synergistic effects, 380 (21.94%) were antagonistic, and 1,264 (72.98%) were additive (Table 3). In contrast, found that synergism was the most common type of pharmacodynamic pDDI (65.1%), followed by antagonism (34.9%) (Faisal et al., 2018). In contrast, Barot et al. (2015) found that pDDIs synergized the most (65.6%), followed by addition (22.4%), and antagonism (29.12%) (Barot et al., 2015).

Among the pharmacokinetic interactions recorded, there were 492 pDDIs (19.65%) of the total. Of these, 6.22% were related to absorption, 35.57% to distribution, 61.59% to metabolism, and 1.63% to excretion. An Indian study found that metabolism is the most common pharmacokinetic type (73.0%). Still, absorption (13.5%) and excretion (13.5%), the second and third types, respectively, were different (Barot et al., 2015). Similarly, 86 (11.6%) of the subjects in an Iraqi study were determined to have metabolism, 33 (4.5%) to have absorption, and 25 (3.4%) to have elimination (Abdul-nabi et al., 2021). Conversely, out of 553 pharmacokinetic studies (26.76 total), 217 (10.50%) focused on absorption and 145 (7.01%) on metabolism (Patel et al., 2014).

The study found that moxifloxacin and amiodarone shared a single “X” pDDI. According to Aydin and Aydin (2024)a single patient experienced pDDIs while taking levofloxacin and amiodarone (Aydin and Aydin, 2024). A Serbian study of six participants noted the interaction between amiodarone and ciprofloxacin (Kovačević et al., 2017). Compared to the prior study, this one had a smaller sample. Several drugs interact with quinolones. According to Kang et al. (2001), quinolones can lengthen the QT interval by blocking cardiac potassium voltage-gated channels (Kang et al., 2001). Therefore, it is preferable to avoid taking this medicine at the same time as other drugs that can increase the QT interval if possible. Regarding quinolones, cardiac arrhythmias, and QT prolongation, moxifloxacin, levofloxacin, and ciprofloxacin are the most commonly associated (Gorelik et al., 2019). Additionally, fewer cases of drug interactions were inappropriate since clinical pharmacists were on staff at the hospital where the study was conducted.

In this study, bleeding was the most common pDDIs observed in class D, with 92.4%. Similar results were indicated by the aspirin and heparin/low molecular weight heparins, which enhance anti-thrombosis efficacy in acute ischemic stroke patients (Powers et al., 2018). Within class C, concurrent usage of ramipril with aspirin was common (281, 15.0%), followed by drugs that increase bleeding risk (aspirin with clopidogrel (6.7%), and rosuvastatin with ticagrelor (6.1%)).

Based on our findings, ramipril and aspirin were the most prevalent interacting pairs (281/2,504, 11.2%), followed by aspirin and ticagrelor (232/2504, 9.2%) and aspirin with clopidogrel (125/2504, 5.0%). Aswani et al. (2016) found that aspirin-ramipril (41%) (Aswani et al., 2016). Devarashetty et al. (2020) found that the most common interacting pair was aspirin and clopidogrel (54%), followed by aspirin and ramipril (41%) (Devarashetty et al., 2020). Another study conducted by Al-Amin et al. (2012) found that the most prevalent interaction was between aspirin and clopidogrel (46.85%) (Al-Amin et al., 2012). Aspirin irreversibly reduces prostaglandin production, reducing the therapeutic benefits of all antihypertensives, including ACEIs (Banerjee, 2012).

Akbar et al. (2021) found that aspirin with enoxaparin (n = 229) was followed by clopidogrel with enoxaparin (n = 214) (Akbar et al., 2021). In addition, a study performed by Hamadouk et al. (2023) noticed that clopidogrel with pantoprazole and ceftriaxone with furosemide (n = 18.7.6%) were the most common in class C, followed by clopidogrel with aspirin (7.2%) (Hamadouk et al., 2023). Prostaglandins are essential for the pharmacological action of ACEIs. In contrast, aspirin irreversibly inhibits the synthesis of prostaglandins, thereby attenuating the therapeutic effects of nearly all antihypertensives, including ACEIs (Banerjee, 2012).

Dual antiplatelet therapy (DAPT), indicated by the combination of aspirin and a P2Y12 is prescribed by the European Society of Cardiology (ESC) and the American College of Cardiology (ACC) for the primary and secondary prevention of coronary heart disease (CHD), as well as for the maintenance of stent patency (Byrne et al., 2024; Kumbhani et al., 2025). Aspirin inhibits platelet activation through the TXA2 pathway. In contrast, clopidogrel operates by blocking the P2Y12 receptor, resulting in a synergistic antithrombotic effect (Brunton et al., 2018). Pharmacodynamic interactions between aspirin and clopidogrel cause this drug interaction. The physician needs to keep an eye out for the possibility of bleeding because this combination therapy falls under risk category C. The dual approach is beneficial because it may offer additional benefits beyond monotherapy.

Clopidogrel and proton pump inhibitors (PPIs) are frequently administered together because PPIs minimize the risk of bleeding in the gastrointestinal tract in individuals who are at high risk (Levine et al., 2016). Pharmacodynamic interactions between aspirin and clopidogrel cause this drug interaction. The physician needs to keep an eye out for the possibility of bleeding because this combination therapy falls under risk category C. The dual approach is beneficial because it may offer additional benefits beyond monotherapy.

Clopidogrel and proton pump inhibitors (PPIs) are frequently administered together because PPIs minimize the risk of bleeding in the gastrointestinal tract in individuals who are at high risk (Levine et al., 2016).

In this study, a patient with pDDIs who was taking clopidogrel and pantoprazole was identified as belonging to risk category C, which indicates that a medication modification is necessary following an evaluation of the risks vs. the benefits. The FDA recommends avoiding the use of clopidogrel and omeprazole or esomeprazole together (U. S. Food and Drug Administration, 2018). According to the guidelines, patients who are on DAPT and are at high risk for gastrointestinal bleeding should be given PPIs, specifically pantoprazole or rabeprazole, because these medications have a lower affinity for CYP2C19 (Dunn et al., 2012). Even though this interaction was anticipated to be in risk category C in the current study, insufficient information is available to determine whether it is clinically significant.

In this study, 44.2% were noted in the alimentary tract and metabolism, 43.03% in the cardiovascular system, and 10.98% in the organs responsible for blood and blood-forming organs. On the contrary, a Jordanian study found comparable results: 46.6% (were observed in the cardiovascular system, 25.5% in the digestive tract, and 15.9% in the organs responsible for blood and blood-forming organs (Nusair et al., 2020). The cardiovascular system accounted for the most significant percentage of ATCs (372 [51.24%]) in this analysis, followed closely by blood and hematopoietic organ medicines (288 [39.67%]) (Fettah et al., 2018).

The most common organ system was the cardiovascular system (85% of the total), followed by the digestive tract and metabolism (79.6%), and finally, blood and blood-forming organs were the least common (53.1%) (Al-Qerem et al., 2018). In contrast, the Netherlands reported that 1,223 organs were involved in blood formation, 943 antibacterial drugs for systemic use, 582 drugs for the cardiovascular system, and 12 drugs used to treat diabetes (Askari et al., 2013). A study conducted in Brazil revealed that the alimentary tract and metabolism were analyzed the most frequently (25.7%), followed by “anti-infectives for systemic use” (9.9%), “cardiovascular system” (13.1%), and “nervous system” (12%) (Lima and Cassiani, 2009).

In our study, age, comorbidities, and length of hospital stay were statistically significantly associated with pDDIs risk rating. Similar to Pejčić et al. (2019), which shows that age, diabetes mellitus, and hypertension elevated the prevalence of pDDIs in ACS patients (Pejčić et al., 2019). Sahoo et al. (2024) also showed that being female, using fixed-dose combinations, and having diabetes mellitus, high blood pressure, and dyslipidemia all had a beneficial effect on clinically significant pDDIs (Sahoo et al., 2024). Moreover, Khan and Hussain (2024) find that age, duration of hospital stay, and the quantity of medications elevate the incidence of substantial pDDIs (Khan and Hussain, 2024). As well as Akbar et al. (2021) demonstrated that patients experiencing myocardial infarction (n = 150, 96.2%) who were prescribed more than 12 medications (n = 45, 88.2%) exhibited an increase in pDDIs (Akbar et al., 2021).

In a multiple logistic regression study found that the STEMI patient, length of hospital stay, and number of drugs provided were significantly associated with the occurrence of pDDIs analysis. pDDIs were not positively predicted by age, BMI, hypertension, diabetes, dyslipidemia, IHD, and Killip class, while STEMI in Dand X was associated with a longer hospital stay and more drug administration, which were significantly higher in classes B and C. Similar to our findings, the number of comorbidities did not affect the prevalence of pDDIs, as reported by Akbar et al. (2021), who found that comorbidities cannot predict pDDIs, whereas only STEMI and the number of drugs greater than 12 positively predict pDDIs (Akbar et al., 2021). Likewise, an Egyptian study conducted by Raslan et al. (2018) found that the comorbidities and age cannot predict pDDIs (P = 0.124 and P = 0.167) compared to drugs >7 drugs and hospital stay >7 days (Raslan et al., 2018), in agreement with my results. Age did not positively predict the presence of pDDIs as reported in three previous studies (Raslan et al., 2018; Khan et al., 2019; Akbar et al., 2021).

In the present study, the length of hospital stay was negatively predicted by pDDIs. In the same line as Sahoo et al. (2024) demonstrated that the length of hospital stay was not a predictor of pDDIs among ACS patients (Sahoo et al., 2024). Akbar et al. (2021) also showed that the length of hospital stay was not positively associated with predicted pDDIs (Akbar et al., 2021). Furthermore, there was no positive association between the number of drugs administered and predicted pDDIs. In contrast to our study, conducted in Ethiopia (Endalifer et al., 2023), India (Shetty et al., 2018), Uganda (Lule et al., 2024), and Egypt (Raslan et al., 2018).

Patients in groups D and X reported fewer anticipated pDDIs despite receiving a higher quantity of drugs, in contrast to groups B and C. This indicates that polypharmacy alone may not consistently increase the risk of pDDIs. One possibility is that clinical pharmacy interventions, including active prescription evaluation and modification, may have reduced potential interactions in these high-risk populations. Moreover, despite higher medication costs, the implementation of safer, more standardized treatment protocols has unequivocally reduced observed interactions. Software and drug interaction screening can hamper the detection of pDDIs. Systems display all interactions or only clinically significant ones.

The principal strength of this study is its prospective design. At the same time, its limitations include being conducted at a single center, which may limit the generalizability of the findings to other contexts. Patients treated mainly in outpatient clinics were excluded, limiting the findings to hospitalized ACS patients. We used Lexi-Interact to find pDDIs. It simply looks for potential interactions, not true adverse clinical effects. The principal investigator identified all pDDIs without the assistance of a second reviewer, thereby compromising the reliability of the data.

## Conclusion

pDDIs were more prevalent among the ACS hospitalized patients at Assiut University Heart Hospital. The pDDIs detected had moderate severity. Aspirin had the most potential for pDDIs, especially when combined with ticagrelor. These pDDIs may significantly impact treatment success, hospital stay, and healthcare outcomes.

## Data Availability

The original contributions presented in the study are included in the article/supplementary material, further inquiries can be directed to the corresponding author.
